# District level external quality assurance (EQA) of malaria microscopy in Pakistan: pilot implementation and feasibility

**DOI:** 10.1186/1475-2875-10-45

**Published:** 2011-02-17

**Authors:** Muhammad Amir Khan, John D Walley, Muhammad Arif Munir, Muhammad Aslam Khan, Nayyar Ghias Khokar, Zarfishan Tahir, Athar Nazir, Nazia Shams

**Affiliations:** 1 Association for Social Development, Islamabad, Pakistan; 2 Nuffield Centre for International Health, University of Leeds, UK; 3 Directorate of Malaria Control, Islamabad, Pakistan; 4 Institute of Public Health, Lahore, Pakistan

## Abstract

**Background:**

Prompt, quality assured laboratory diagnosis is key to effective malaria case management and control, especially since the introduction of the more expensive artemisinin combination therapy (ACT). The malaria programme and its non-government partners, on the basis of WHO recommended Lot Quality Assurance methods, have developed a district level external quality assurance (EQA) system. This study was designed to assess the feasibility, under programme conditions, of an integrated district level external quality assurance and supervision approach for malaria microscopy.

**Design and Methods:**

A prospective study conducted over seven months period (May-November 2007). In addition to the standard WHO EQA elements, three operational innovations were introduced, with the a district laboratory supervisor: a) onsite re-checking of slides, b) in ensuring uninterrupted availability of laboratory reagents and supplies at diagnostic centers, and c) supervision of administrative and technical components. The quantitative data for the study came from the service records/documents, whereas the qualitative data came from the key informant interviews.

**Results:**

During the seven month period in four districts, a total of 8,118 slides were examined of which 209 (2.6%) were found positive for malaria parasites (slide positivity range between1.6% to 6.0%). The District Laboratory Supervisors in four districts reexamined a total of 1,770 slides (22%). The proportion of slides found discordant ranged from 0.5% to 1%. The quality of smear preparation was found acceptable in 73% slides.

**Conclusions:**

A district-based EQA, based on lot quality assurance methods was implemented, using context-specific operational guidelines, tools and training modules, and other inputs from the malaria control programme and partners. This EQA and supervision approach was found to be feasible and acceptable to those involved. Further study is required on the microscopy quality and cost-effectiveness of adding external quality assurance and supervision to district malaria microscopy services.

## Background

Malaria remains a leading cause of morbidity and mortality world-wide with one million deaths occurring annually [1]. Prompt, reliable laboratory diagnosis is recognized as an important component of effective malaria case management and control [2,3]. Strengthening laboratory diagnosis should help reduce malaria morbidity and mortality. This has become particularly important since the introduction of the more expensive artemisinin combination therapy (ACT).

The Pakistan national strategy for malaria control relies on a network of laboratories that provide malaria microscopy. If the laboratory diagnosis is unreliable, the malaria case management will be poor. Therefore, quality assurance of malaria microscopy services is essential for early diagnosis and prompt treatment of malaria cases - a key component of the WHO Roll Back Malaria (RBM) strategy. Technical resources for performing quality assurance of malaria microscopy are available, including those developed by the WHO [4,5]^. ^However, these international guidelines focus mainly the technical aspects. These technical guidelines are useful reference material for method selection, implementation, and issues and interpretation that are encountered in an external quality assurance (EQA) Programme.

External quality assurance of malaria microscopy, through a countrywide network of more than 1,100 diagnostic centers, is a priority challenge being faced by the national malaria control programme (NMCP). The national malaria control programme is agreed to provide equipment, training and material inputs for improved functioning of the laboratories at the diagnostic centers. The Programme is receiving public funding for countrywide implementation of roll back malaria interventions including case management strengthening with quality assured microscopy. However, the programme lacks context-sensitive operational guidelines and tools for implementing external quality assured malaria microscopy in Pakistan.

The current study has been designed to evaluate context-sensitive operational guidelines (adapted from international guidelines [4]) and tools for district level external quality assurance of malaria microcopy in Pakistan. The dissemination of this experience would also help other countries in the region to develop their operational strategies for district level quality control of malaria microscopy.

## Methods

The Association for Social Development, Pakistan and Nuffield Centre for International Health, United Kingdom working in partnership with the National and Provincial Malaria Programme, Punjab, organized a small technical working group to develop a set of context sensitive operations and tools for implementing district-based external quality assurance, under programme conditions. The design was based on WHO recommended Lot Quality Assurance (LQA) approach to external quality assurance. The approach allows a small number of slides to be selected for quality control with a sound statistical basis for interpreting the results [4]. The uniform sample size method was recommended to simplify the Lot Quality Assurance (LQA) for routine programme circumstances. The required sample size of 24 slides per quarter was calculated on the basis of assumed average values for each laboratory: a testing volume of 1,000 - 2,000 slides per year, a slide positivity rate of 10%, and sensitivity of 80%. The same working group then developed a training package to enable the district laboratory supervisor for carrying out the external quality assurance activities, as per agreed guidelines.

To implement the new guidelines, each of the four selected districts, as per local circumstances, designated a senior microscopist as district laboratory supervisor. In two of the four districts the selected district laboratory supervisors were enabled and given responsibility for ensuring quality of both malaria and TB (AFB) microscopy, whereas, in other two districts the district laboratory supervisor was responsible only for quality of malaria microscopy.

The malaria external quality assurance enabling inputs in all these districts included: a) training each district laboratory supervisor, at provincial reference laboratory, for ten days on malaria microscopy and then for four days on external quality assurance of malaria microscopy. The core ten days training was on national programme materials, whereas the four-day training course on quality assurance was on a new training package developed to impart the required knowledge and skills. The training methods included reading of text supplemented with role plays, exercises and discussions (see external quality assurance guidelines on web link: http://www.asd.com.pk/products.htm), b) providing district laboratory supervisor with a motorbike, petrol cost and field operating expenses, c) supervising district laboratory supervisor work through his regular interaction with District Malaria Focal Person.

The three key set of innovations introduced in this district based external quality assurance model were: a) district laboratory supervisor on-site rechecking of slides and technical support to the facility staff, and combining smear assessment and slide reexamination for quality assurance b) district laboratory supervisor defined role in uninterrupted availability of laboratory reagents/supplies at diagnostic centers, c) administrative and technical supervision of district laboratory supervisor by District Malaria Focal Person and provincial reference laboratory respectively.

The enabled district laboratory supervisor planned and visited each of 10 - 15 diagnostic centers in his district, on monthly basis. During each monthly facility visit, the district laboratory supervisor was expected to: a) review, replenish (where needed) and record the laboratory material inputs, b) assess the laboratory arrangements and procedures, c) ask the facility doctor to select and give a sample of slides for reexamination, d) reexamine on-site the given sample slides, and record results in a newly designed form, e) provide his feed back and suggestions to the facility doctor and laboratory staff, and f) retain the reexamined slides for storage at district level.

The distribution of reagents and laboratory supplies to facilities was mainly through two mechanisms: a) periodic distribution to each diagnostic center from the district health office/store, and b) monthly onsite replenishment by district laboratory supervisor, during his external quality assurance visit.

In each district, the external quality assurance related records and concordant slides collected from the facilities, as well as required equipment and supplies were kept at the district level external quality assurance center. The concordant slides were kept until the provincial reference laboratory staff reexamines a sample of these slides. On monthly basis, all the discordant slides (i.e. where the supervisor's results do not agree with the laboratory staff results) found in the district were packed in a wooden box and sent to the provincial reference laboratory, through locally available courier service. These discordant slides were examined by an experienced microscopist at provincial reference laboratory and feedback was send back to District Malaria Focal person through regular mail system.

Two main officers supported and supervised the district external quality assurance activities were: a) District Malaria Focal Person - mainly responsible to ensure: the visits of district laboratory supervisor, as per agreed plan; the required records and slides were kept; and reagents and other laboratory supplies were distributed in a timely way, and b) the provincial reference laboratory - mainly responsible to ensure: the discordant slides received from districts are reexamined and feedback is provided within the quarter; the visit of each district (2 - 4 times per year) to supervise DLS working and trouble-shoot at identified problematic facility(ies).

The initial seven-month (May - November 2007) implementation in selected four districts was studied to assess the feasibility, under programme conditions, of the district level external quality assurance for malaria microscopy. The quantitative data for the study came mainly from the records/documents, whereas the qualitative data came from the key informant interviews.

The records reviewed for the study included: a) EQA-01 forms filled by the district laboratory supervisor during each facility visit, and b) EQA-02 forms filled every month for each district, by district laboratory supervisor and then provincial reference laboratory staff, and c) monthly field visit plans for external quality assurance in four districts. The data extracted from the records were computerized and then analysed (Microsoft Excel was used for data entry and SPSS for data analysis).

The key informants interviewed for the study included: a) District Laboratory Supervisors (4), District Malaria Focal Persons (4), Regional Coordinators (2), Laboratory technicians (12), provincial staff (2), and District health store person (4). Trained qualitative researchers conducted key informant interviews, after obtaining informed consent, using especially designed interview schedules. These researchers then transcribed the data, with the help of a senior researcher. The senior researcher then collated the transcribed data into themes and groups of information. A group of researchers and programme staff then jointly interpreted the collated quantitative and qualitative data to draw inferences.

## Findings

### District laboratory supervisor (DLS) Identification and training

A senior laboratory technician was identified as District Laboratory Supervisor in all four districts from the available laboratory personnel. The Provincial Malaria Control Programme, through district implementation planning exercise, facilitated the transparent selection of suitable candidates in each of four districts (see Malaria district implementation guidelines. Go to web link http://www.asd.com.pk then click on 'products" then click on "EQA Malaria").

These trainings at the provincial reference laboratory were arranged through project resources, with the facilitation of provincial malaria control programme. All the four district laboratory supervisors attended these province-level trainings, without any problem related to for example: permission from the district office; and modest but replicable arrangements for accommodation, per-diem and travel. The reaction evaluation of the district laboratory supervisor training event showed satisfaction of trainees and trainers with the training contents, methods and materials.

### District Laboratory Supervisor's monthly visit - plan and conduct

District laboratory supervisors were assigned the task to visit each microscopy centre in their respective districts on monthly basis. The district laboratory supervisors regularly prepared monthly visit plans, got these endorsed by the respective district health office, and kept the record.

The health facilities were found informed in-advance about the visit. Deviations from the approved plan/schedule were found minimal. The facility staff, the district laboratory supervisors and the district managers found the informed visit approach useful and efficient.

*"Prior to my visit to microscopy centres, I every month send my visit schedule to the centres by postal service. In addition I used to call them before my visit. This approach has been quite helpful, as staff and relevant records were available whenever I visited the health facilities" (*District laboratory supervisor, *Jhang)*

The number of microscopy centers in a district varied according to the size and profile of the district. The average number of microscopy centers in a district was 11 (range: 8 - 21). Each month, at least 75 percent of the microscopy centers were found visited by district laboratory supervisor. However, in districts with more than ten microscopy centres, the district laboratory supervisor reported difficulties in covering all centres every month.

*I was not able to cover all the health facilities due to two reasons. Firstly these were widely scattered and secondly fixed POL was not adequate to cover all diagnostic centres in a larger district like Jhang". *(District laboratory supervisor, Jhang)

An average travel time for a round trip to the facility ranged between 1 - 4 hours. Normally, the district laboratory supervisor used motor bikes for visiting the health facilities, except in some situations where public transport was preferred for far off health facilities.

*"Some facilities in my district are far with poor roads. Two of the centres were almost 105 km away. It was very difficult to go by motor bike, so I traveled by bus*. (District laboratory supervisor, *Dera Ghazi Khan*)

*"For some microscopy centres it was not possible for me to return on the same day and I had to spend a night at the facility"*. (District laboratory supervisor, Bhakkar).

The District Malaria Focal Person and District Laboratory Supervisors in the two larger districts suggested visiting microscopy centres on quarterly basis, instead of monthly. They also perceived the programme logistic support important for continuing the exercise.

### Reagents preparation and distribution

The preparation of Giemsa stain at district level, by district laboratory supervisor, was found feasible. None of the facility laboratory staff complained about the quality of the stain supplied. The district laboratory supervisors were found able to administer positive and negative controls for quality of the stain prepared.

Most of the district laboratory supervisors found the onsite replenishment of reagents and supplies helpful.

*"I maintain a stock register to record the issuance of reagents/supplies to the facilities. I get these transactions signed by the facility staff" *(District laboratory supervisor, Bhakkar)

The stock out reports during the seven months reviewed and electronically compiled showed less than 10% of facilities reporting stock out of one or more malaria laboratory supplies and reagents (Go to http://www.asd.com.pk then click on 'products" then click on "EQA Malaria" for guidelines on stock out data etc.) (see Table 1)

**Table 1:** Overall quality of smear preparation during seven months

No of Facilities	Stock out (one or more malaria laboratory supplies/reagents)													
	
	May		June		July		August		September		October		November	
	
	#	%	#	%	#	%	#	%	#	%	#	%	#	%
48	4	8.3	3	6.2	3	6.3	4	8.3	2	4.1	2	4.1	4	8.3

### Onsite reexamination and support

**Figure 1:**
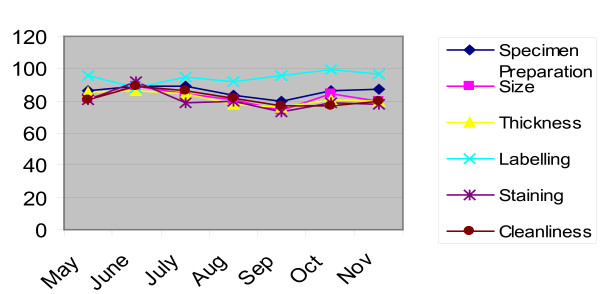
Overall quality of smear preparation during seven months

A total of 1770 slides were reexamined at the facilities, which was 22% of the total 8118 slides prepared during the seven month period. The proportion of slides found discordant (i.e. where the supervisor's results do not agree with the laboratory staff results) ranged between 0.5 - 1%. The proportion of discordant slides does not show any clear time-trend. The quality of smear preparation (as assessed on the basis of six key characteristics i.e. size, shape, thickness, labeling, staining, and cleanliness) was found acceptable in about 73% (i.e.1,293) of these slides (Figure 1) (ref: web link http://www.asd.com.pk click on 'products" then click on "EQA Malaria". see guidelines).

Reexamining eight slides at each facility was found feasible and acceptable. The involvement of facility doctors in the external quality assurance process was found helpful in terms of ensuring the "blinding" and enhancing the ownership and inputs of clinicians in the laboratory functioning.

"Once the doctors were enabled and involved, their sampling practices became more uniform and as per national guidelines" (District laboratory supervisor, Jhang).

The average time required to recheck the slides at a health facility was about one and a half hours. The non-peak hours (i.e. before 10.00 am and after 12.00 pm) were preferred. The district laboratory supervisors generally found the facility staff (laboratory person and doctors) cooperative and responsive to the laboratory quality assurance process.

The laboratory staff found the onsite rechecking of slides helpful for enhancing the skills and improving the practice of malaria slide preparation.

*"Onsite reexamination of slides helped improving my laboratory work. Now I prepare better slides and give correct MP results". *(Laboratory staff, Layyah)

The involvement of District Malaria Focal Person, as an administrative supervisor, was found helpful in district laboratory supervisors' field activities and external quality assurance at health facilities.

*"The DLS has followed the *external quality assurance *guidelines to maintain records and cross-check slides". (*District Malaria Focal Person, Dera Ghazi Khan)

The involvement of provincial reference laboratory, as technical supervisor, was found helpful for cross-checking of the discordant slides. However, human resource and logistic challenges were faced to arrange regular visits of the provincial reference laboratory staff to these districts.

*"Difficulties in making travel and accommodation arrangements limited our opportunity to supervise the external quality assurance in districts. Furthermore, guidelines and tools for conducting the district supervisory visit need more attention"*. (Provincial reference laboratory staff, Punjab)

In four districts, 209 (2.6%) of the total of 8,118 slides examined were found positive for malaria parasites (MP). This slide positivity rate of 2.6% (range: 1.6% to 6%) is thirty-two times higher than the 0.08% reported for the Punjab province in the year 2006.

In the four districts a total of 5670 patients were treated for malaria, out of which only 209 (3.6%) were microscopically confirmed malaria cases. The case management practices have not been inline with the programme policies.

## Discussion

Lot Quality Assurance method is considered a preferred alternate to the conventional approach to rechecking slides (i.e. all positive and 5 - 10% negative) for quality assurance. This gives valid results for high and low volume laboratories, without unnecessarily increasing the work load. After Thailand and Philippines, other Asian country programmes are also considering switching to the Lot Quality Assurance approach [4]. The scaling-up of the approach can be facilitated by developing and making available a set of more robust implementation guidelines and tools. The Pakistan initiative is a step towards addressing the development needs for wider use of WHO recommended approach.

The uniform sample size approach, rather than complex calculation of sample size for each facility based on slide load, positivity etc., was used mainly to simplify the operations at ground level. This uniform sample size approach has widely been used for external quality assurance of sputum microscopy in tuberculosis e.g. in Ghana [6] and Mexico [7], but there is little information about this approach being used for external quality assurance of malaria microscopy except in Philippines [4].

Involvement of facility doctors in laboratory quality assurance process is an interesting experience that needs more attention. In Pakistan, like many developing country programmes, multiple tiers of laboratory staff are not available at district level to manage the sampling and then blind rechecking of slides. The involvement facility doctor, after two-hour orientation, in the blind rechecking made it possible for the quality assurance process to be managed by a district laboratory supervisor. The value of team approach to the laboratory quality assurance process, as indicated in the early experience, need be studied further for wider replication.

There were concerns regarding "quality of blinding" in onsite rechecking of slides. However, early experience of onsite rechecking shows that satisfactory blinding can be maintained, through involvement of facility doctors. The onsite approach found workable as well as acceptable to the facility and district staff need be further assessed for the validity of rechecking process and results.

In this study the overall discordance rate was very low (i.e. between 0.5 - 1%). On contrary studies in Nigeria and South Africa reported discordance of 16.7% and 13.8% respectively [8,9]. The low-discordance results in Pakistan can possibly be due to more experienced and better trained microscopists or a poorly functioning quality assurance. A further study is required to compare the results of off-site and on-site rechecking of malaria slides in Pakistan.

In the present study, each month, the district laboratory supervisor could visit at least 75% of the microscopy centres. This type of onsite rechecking and supervisory visits to microscopy laboratories have not been reported as a routine practice in many developing countries [6,10]. The frequency of district laboratory supervisor's visit to a microscopy centre is an important decision to be made in light of technical requirements and administrative limitations. In cases, where number of facilities or geographic spread makes monthly visit difficult the quarterly visit can be considered as an alternate option. However, quarterly visit option needs assessment before further consideration.

In the study the provincial reference laboratory staff could not be mobilized, mainly due to resource constraints, to visit the districts and supervise the external quality assurance practices. The potential usefulness of regular supervisory visits to the districts, by provincial reference laboratory staff, remains an area to be further developed and assessed in future.

The study also developed and piloted an intervention of onsite replenishment of laboratory supplies and reagents, during district laboratory supervisor visit to the facility. The feasibility of motorbikes with carrier boxes, provided to the district supervisors, may vary in different circumstances (e.g. hilly areas, deserts etc.), and would need assessment in other socio-economic and geographic circumstances.

A good smear does have a role in reducing reading errors, whereas poorly prepared blood films generate artifacts that can be mistaken for malaria parasites [11]. In the current study the quality of smear was found acceptable in about 73% of the slides which is lower then expected. This reflects that more emphasis is required to be given on smear quality dimensions (e.g size, staining, thickness etc.)

In the current study the external quality assurance of malaria microscopy demonstrated a thirty-two times increase in the slide positivity rate when compared with the previous year statistic. The external quality assurance might have contributed in the increased slide positivity rate in four districts. However, further studies are required to understand and explain any possible association in this direction.

The deviant case management (i.e. only 3.6% were confirmed out of the total 5670 treated for malaria), clearly shows that the external quality assurance of laboratory services are not enough to achieve the desired case management practices, unless supplemented with other programme efforts.

The study exercise was mainly to assess mainly the feasibility of implementing a district-level external quality assurance (and related operational guidelines), under programme conditions. The study was not designed to provide a conclusive evidence for effectiveness or scientific efficacy of external quality assurance interventions.

While interpreting these preliminary results and drawing conclusions, we need to keep in mind that seven month period was too short to have a valid results and trends for: a) external quality assurance related reduction of false positive and false negative results, and b) improved quality of slide preparation.

The proposed exercise did not include a detailed cost analysis of adding external quality assurance to district malaria microscopy services. However, review of available costs data revealed that there was a capital cost of about 90,000 Pak rupees (1400 US dollars) to put in place the district external quality assurance of malaria microscopy. This included for an external quality assurance center, a motorcycle, and training of district laboratory supervisor. Also, that adding external quality assurance to the ongoing microscopy resulted in about 50% increase in the direct cost per slide examined (i.e. not including the capital and the staff time costs). In Pakistan, an average direct cost of preparing a slide was about 20 rupees, with 10 rupees added per-slide cost for external quality assurance (US$ 1 = 65 Pak rupees). This 50% increase in direct per-slide cost may appear high, but this is marginal to the high capital and recurrent costs of microscopy services, as well as potential in rationalizing the use of the new more expensive ACT drugs [4]. More detailed costing studies in Pakistan and elsewhere are required to give better understanding of the cost of external quality assurance and enhanced supervision.

This experience also indicates the possibility of integrating the district level external quality assurance of malaria and tuberculosis microscopy. However, the technical and programmatic implications of such integration (including costing) need further careful assessment.

## Conclusion

A district-based external quality assurance, based on lot quality assurance methods, integrated within the district supervisory and facility service was found feasible. The use of context-sensitive operational guidelines, tools and training materials and some other malaria programme inputs helped operationalize this approach. Further evidence is required to assess the effectiveness, e.g. in terms of impact on microscopy quality. and cost-effectiveness of adding external quality assurance and enhanced supervision to district malaria microscopy services.

## Competing interests

The authors declare that they have no competing interests.

## Authors' contributions

MAK being the Principal Investigator contributed to proposal write-up, study design, data analysis, interpretation of results and write-up, JDW being the Co-Principal Investigator contributed in the design, data analysis, interpretation of results and write-up, MAM, as Senior Researcher, had contributed in overall project monitoring and supervision, data analysis and write-up, MAKN as Director National Malaria Control Programme had contributed in the design and conduct of the study, NGK contributed in the field implementation and data collection, ZT contributed to the review of guidelines and monitoring, AN was involved in quantitative data collection and conducting interviews, NS contributed in the data entry and analysis. All authors read and approved the manuscript.
